# AI-Assisted Vision Alarming System for Blind and Vision- Impaired People

**DOI:** 10.3390/s26102929

**Published:** 2026-05-07

**Authors:** Le Chung Tran, Sinh Khai Ly, Rhys Blacklidge, Jonathan Shemmell, Nathan Difford, Daniel Edward Cox, Theresa Harada

**Affiliations:** 1 School of Engineering, University of Wollongong, Northfield Avenue, Wollongong, NSW 2522, Australia; skly@uow.edu.au (S.K.L.); rhysblacklidge4@gmail.com (R.B.); shemmell@uow.edu.au (J.S.); nd945@uowmail.edu.au (N.D.); tharada@uow.edu.au (T.H.); 2 Trans Traffic Survey, 3 Hepburn Way, Melbourne, VIC 3023, Australia; daniel@trafficsurvey.com.au

**Keywords:** blind, vision-impaired, AI, IoT, YOLO, Raspberry Pi 5, DepthAI, ROS 2, SoX, LiDAR, Oak D-Lite, TTS

## Abstract

Navigating through everyday environments, like walking down a sidewalk, which many people often take for granted, is a difficult task for millions of people with vision impairments since it involves sophisticated object detection, depth perception, and situational awareness, all working seamlessly to guide a person through complex surroundings. Many current assistive devices for vision-impaired people are either expensive, information-overabundant, or missing critical information. This paper details our Vision Alarming System (VAS), which can improve the safety for blind and vision-impaired people by providing awareness of both positions and nature of nearby obstacles; thus, assisting users to make decisions to avoid collisions, reduce accidents and casualties, while enhance their experience, independence, and confidence when participating in traffic. VAS is an Artificial Intelligence/Internet-of-Things (AI/IoT)—powered system developed utilizing the cutting-edge Raspberry Pi 5, a Light Detection and Ranging (LiDAR) sensor, and an AI depth camera, operating as different containers in a Docker architecture, and leveraging a Robotic Operating System 2 (ROS 2) backbone. VAS communicates the obstacle detections to users via Bluetooth interface, using the neural Text-To-Speech (TTS) system, namely, Piper, and the Sound eXchange (SoX) technologies. Our proof-of-concept system proves that VAS can be a standalone, open-source, extremely low cost, low power consumption assistive device which can synergistically utilize the cutting-edge AI/IoT technologies to provide blind and vision-impaired users with an appropriate amount of critical information about their surrounding environments.

## 1. Introduction

**Figure 1:**
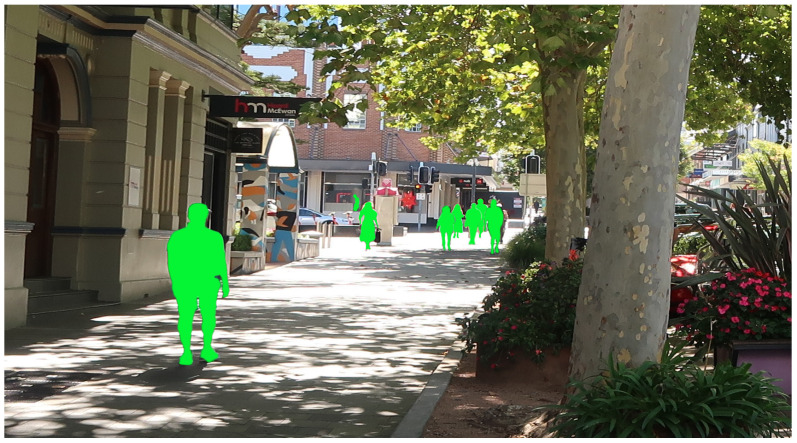
Navigating through everyday environments, like walking down a sidewalk, is a difficult task for millions of people with vision impairments. (People in the picture are obscured for privacy).

According to Vision 20/20 Australia, there are more than 575,000 blind and vision-impaired people living in this country, of whom more than 70% are over 65 years of age and about 66,000 people are blind [1]. Globally, the World Health Organization (WHO) reports that there are around 2.2 billion people with vision impairments, most of whom are over the age of 50 [2]. An aging society along with a dramatic increase in road traffic highlights that blind or vision-impaired people need both smarter and more affordable assistive technologies to help them more actively participate in daily traffic scenarios, as illustrated in Figure 1.

Assistive devices, such as smart canes and smart shoes, have attracted great interests from both researchers and industry. This is partially because guide dogs, which are a common assistive option for many blind and vision-impaired people, are costly to train, yet their working lifespan is limited [3]. Guide dogs might also be distracted in hectic environments where individuals, pets or vehicles appear dynamically in different directions. In addition, commercial products, such as WeWALK Smart Cane Plus with Intelligent Voice Assistant [4] (approximately A$1867) or OrCam MyEye 3 Pro camera [5] (A$4700), are costly for many visually disabled people. These have encouraged more research on both reliable, multi-feature, and cost-effective solutions to better support this group of users.

The rapid development of Machine Learning (ML), Deep Learning (DL), Artificial Intelligence (AI), Computer Vision (CV), and the Internet-of-Things (IoT) over the last decade has considerably promoted the development of smarter assistive devices for the blind and vision-impaired individuals. Reported assistive devices have demonstrated promising abilities in effectively supporting users with vision impairments, and have provided valuable insights for future work, though having some limitations regarding dataset size, compactness, data processing framework, and user-friendliness. For example, in [6], Poggi et al. introduced a wearable assistive system that collected data via a Red, Green, Blue, and Depth (RGBD) sensor, performed object detection and classification, then provided both tactile and audio feedback to its users. Real-time obstacle detection was achieved with a high precision of 97.93%. However, their training dataset size for obstacle classification was limited, which might affect the system ability in detecting a wide range of objects. The authors in [7] presented an image-captioning framework utilizing a Text-To-Speech (TTS) engine, the Visual Geometry Group 16-layer (VGG16) Convolutional Neural Network (CNN) model, and the Long Short-Term Memory (LSTM) network implemented on a Raspberry Pi 4B connected with a No Infrared Filter (NoIR) camera. The model showed high scores in their assessment metrics, but there seemed to be a lack of practical implementations and testing. In [8], P. Mohanraj et al. developed an assistive system consisting of a wearable cap and a supportive stick. The cap carried a Pi camera connected to a Raspberry Pi, leveraging the MobileNet Single Shot Detector (SSD) DL model to perform object detection and a TTS system to output audio feedback to users via earphones. The stick carried an ESP8266 micro-controller, a water sensor to detect slippery areas, an ultrasonic sensor to detect obstacles, and a buzzer to provide sound alerts to the user in case of obstructions. An assistive stick was introduced in [9], utilizing a Raspberry Pi 4, a Pi camera, and an ultrasonic sensor that could detect objects, faces, obstacles, and measure distances, but its alerting sound rule might confuse users in case of many obstacles. There was also a lack of results and discussion on outdoor scenarios.

Some authors added important practical and realistic functions to their assistive systems. For example, Rahman et al. in [10] developed an IoT-assistive system capable of object detection and currency note recognition. The system also used an accelerometer to detect when the user fell and alerts would be sent immediately to the user’s guardian. However, the IoT system, whose importance was not adequately clarified, might make the whole system bulky. In [11], Jivrajani et al. introduced a relatively cost-effective, lightweight, and comprehensive system mainly consisting of a Raspberry Pi 3B+, a Global Positioning System (GPS) module to send the location of the user to an application where the guardian could observe, a Global System for Mobile Communications (GSM) module to send alert messages, an ultrasonic sensor for obstacle detection, a water sensor for slippery area detection, a heartbeat sensor, a Pi camera for object and currency notes detection utilizing a custom You Only Look Once (YOLO) version 5s model, as well as LED indicators and timing features for bus timetables. However, the range of obstacle detection was relatively short (up to 1.7 m), highlighting the need of improvements in future work. Utilizing smart phones for assistive purposes was another promising idea by A. N. Krishna et al. in [12]. Their proposed system was developed as an application on a common smartphone that communicated with users via a Chatbot. The system performed mask detection (based on the SSD algorithm), object detection (based on the custom YOLOv4-tiny model), distance estimation, and speech feedback generation. Regardless of a high accuracy in detection tasks, this system must rely on the stability of its wireless connection for data processing at a remote server, and the number of object classes in its training dataset was limited.

Previous works have demonstrated the suitability of embedded devices like the Raspberry Pi and sensory devices in developing visual assistive systems thanks to their compactness, low power consumption, and ease of integration. Valuable insights in leveraging TTS systems and DL models such as YOLOs for object detection and feedback generation purposes were also provided. Some authors even compared the performance of different YOLO models [13], suggesting that tiny models might be more suitable for real-time applications when the detection speed is prioritized. Many researchers also preferred to build their custom YOLO models or other object detection models for higher accuracy and precision [14]. On the other hand, as indicated in [15], to enhance users’ experience, an assistive system should be able to provide clear voice and/or tactile feedback. It should be lightweight and compact to mitigate users’ discomfort and social stigma. Adjustability and modularisation are other noteworthy considerations, while practical experiments are significant to evaluate the system performance and efficiency. Cost-effectiveness is also a crucially important factor in popularising the system among vision-impaired users. Unfortunately, some of these considerations were missing in several previous works.

Building on previous findings, this paper proposes our Vision Alarming System (VAS)—a portable, standalone, reliable, affordable, and real-time assistive solution for vision-impaired people. The system combines the proven advantages of the Raspberry Pi and the neural TTS system, explores the ability of new sensors which are the TF Mini-Plus Light Detection and Ranging (LiDAR) sensor and the Oak-D Lite AI camera. The system utilises the YOLOv4-tiny model, ROS 2 middleware, and Docker containerisation. VAS is suitable for both indoor, outdoor navigation scenarios and dark conditions. Our research team involves a visually impaired member, allowing us to develop a fit-for-purpose solution for vision-impaired people. A qualitative comparison between VAS and previous work, with important criteria such as Internet independence and lightweight/compactness, is shown in Table 1, which suggests that VAS is a good combination of mobility, efficiency, and reliability among the developed assistive technologies.

The main contributions of this paper are summarized as follows.

(1) The paper proposes a fit-for-purpose, cost-effective, standalone solution to assist vision-impaired people in daily traffic participation. The system essentially plugs and plays and does not require any Internet connection, edge computing, or online AI processing. To the best of our knowledge, our system is the forefront device from this comprehensive perspective.
(2) The paper develops the first system comprehensively combining all technical components, namely TF Mini, Oak-D Lite, YOLO, TTS, Docker, and Raspberry Pi. Although the use of cameras, TTS or YOLO has been explored in literature, to the best of our knowledge, none of the existing assistive devices has comprehensively combined all of these technical components. Our system can simultaneously perform object detection, obstacle/object distance measurement, and voice feedback generation. The scalability of the system was considered by leveraging Docker, which has not been implemented in many previous works.
(3) The paper proposes an efficient approach for the fusion of the information retrieved from multiple sensing sources with different rates, including the distance information from the TF Mini and distance, depth, and object classification information from the Oak-D Lite in a reliable and optimized way. As shown in the examples later in this paper, VAS can still detect objects accurately even when the Oak-D Lite does not work due to, for instance, the dark conditions or when one sensor (e.g., TF Mini or Oak-D Lite) freezes. This feature plays an important role in the safety of the users in daily traffic participation.
(4) The paper proposes the design of TTS strategies with suitable Quality of Services levels and optimal TTS message structures, carrying the most critical information to provide fast, reliable, but not excessive notifications to the users. To provide sufficient but not overabundant information, the working range is split into critical and non-critical ranges to prioritize the most urgent obstacles. To the best of our knowledge, VAS is the first system that possesses this feature.
(5) The paper develops a novel grouping algorithm where a group of similar and close objects can be notified to the users by just one brief notification, rather than excessive, individual notifications. This is especially helpful in clustered environments. To the best of our knowledge, our system is the first system facilitated by such an algorithm.

The remaining parts of this paper are organized as follows. Section 2 introduces the system design of VAS, including hardware selection and software development. Section 3 explains our experimental setup, analyses experimental results, and provides relevant discussion. Section 4 presents the conclusion and suggests some potential improvements in future work.

## 2. System Design

### 2.1. Hardware Selection and Configuration

**Figure 2:**
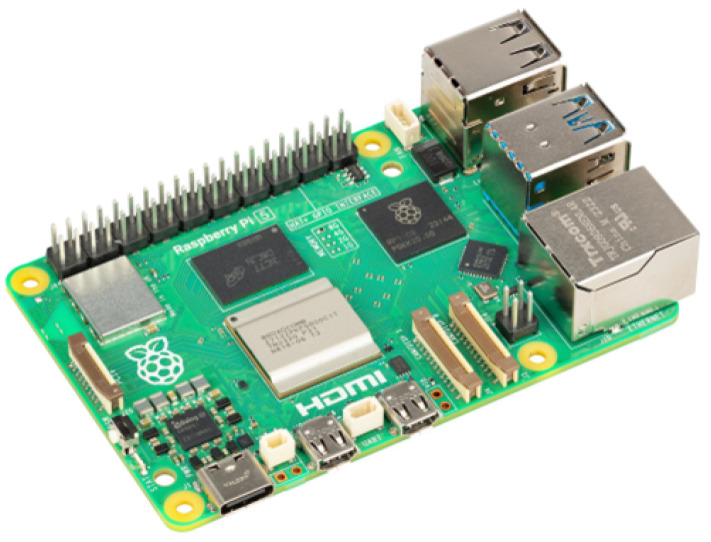
Raspberry Pi 5 single-board computer (image courtesy of Raspberry Pi) [16].

The single-board computer Raspberry Pi 5, as shown in Figure 2, is selected as the central processing board of VAS. With the ARM Cortex-A76 2.4 GHz processor and 8 GB RAM, it can handle multiple parallel tasks of VAS, such as processing of object detection and distance calculation, together with TTS feedback generation [16]. Its USB ports enable an easy configuration via mouse and keyboard, while the dual 4Kp60 HDMI^®^ display output with High Dynamic Range (HDR) helps visualize the results during indoor and outdoor experiments [16]. Low power consumption is another important criterion that makes the Raspberry Pi 5 suitable for assistive systems supporting vision-impaired users who may have to rely on the system for a long outdoor travel.

**Figure 3:**
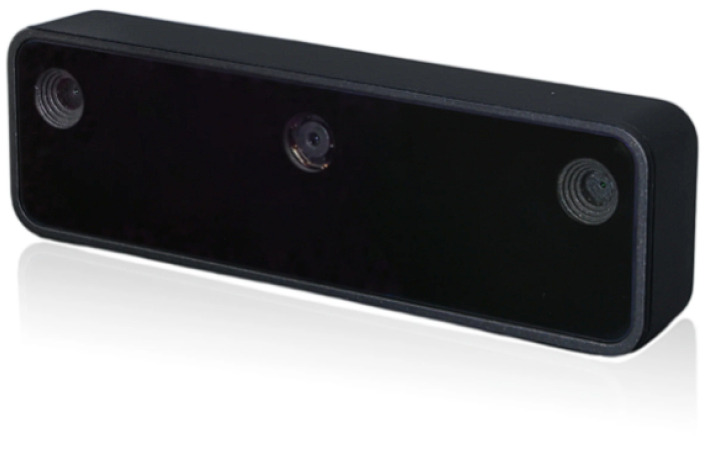
Luxonis Oak-D Lite robotic perception camera [17].

The Luxonis Oak D-Lite AI camera [17] (or Oak-D Lite for short), as shown in Figure 3, is a powerful AI-powered stereo camera equipped with the Myriad X VPU (Vision Processing Unit) offering 4 Trillion Operations Per Second (TOPS) of processing power, whose ability will be leveraged in VAS. It is designed to run DL models, which, in case of VAS, is the YOLOv4-tiny model for object detection tasks. The camera consists of three smaller on-board cameras, namely a 13 MP color camera in the middle and two stereo depth cameras offering below 2%, 4%, and 6% absolute depth errors within 3 m, from 3 m to 6 m, and from 6 m to 8 m, respectively [17], making it a suitable choice for tracking mobile 2D and 3D objects with a reliable distance of up to 8 m [17]. The camera’s small dimension of 91 mm × 28 mm × 17.5 mm and the maximum power consumption of around 5 W [17] suggest that it might be a good option for portable assistive systems like VAS. The Oak D-Lite camera can further bring considerable values to VAS thanks to its auto-focus variant. This variant can clearly recognize both close objects (at distance from 8 cm to 50 cm) and distant objects (at distance of 50 cm and beyond) [17]. In general, the Oak D-Lite camera is a relatively cost-effective but computationally powerful option, allowing VAS to balance between computational capability and manufacturing expense.

**Figure 4:**
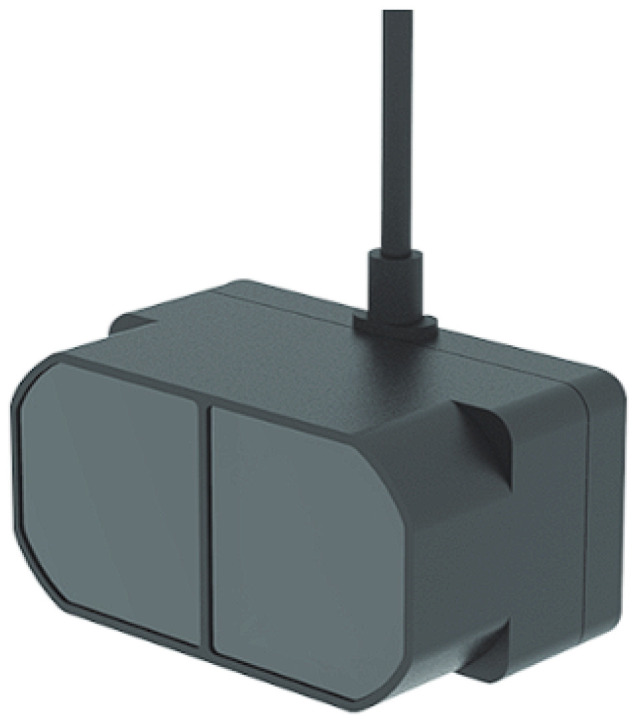
TF Mini-Plus LiDAR sensor [18].

Figure 4 describes the TF Mini-Plus LiDAR (or TF Mini for short), which is a Time of Flight (ToF) sensor integrated into VAS, providing short-to-medium range distance measurements. It has a Field of View (FoV) of 3.6°, an operation range of up to 12 m, with a high accuracy of 5 cm in the range 0.1 m–5 m and around 1% at further distances [18]. Its frame rate is flexible between 1 and 1000 Hz [18], allowing for different applications. The wide operating temperature range from −20 °C to 60 °C [18] makes it a suitable choice for the everyday navigating application of VAS. In addition, its small weight of roughly 12 g and small dimension of 35 mm × 18.5 mm × 21 mm [19] enable an easy integration into VAS. Main technical specifications of the TF Mini are summarized in Table 2.

The TF Mini provides a centimeter-level precision for obstacle detection within its range (up to 12 m) with a distance resolution of 1 cm [19]. This is crucial for VAS to detect obstacles directly in front of the user. In addition, the sensor can publish distance data at a rate of 10 Hz (adjustable). This ensures that even fast approaching objects (like vehicles or bicycles) can be detected in time to warn the user. Moreover, the sensor is designed for a minimal power consumption (550 mW normally or under 100 mW in its Low Power Consumption Mode) [19], making it a useful and energy-efficient addition to VAS. Furthermore, the TF mini uses its own laser signal to calculate distances to objects; thus, it is independent of lighting conditions of the environment. This is an important ability that VAS will leverage in assisting users in dark indoor and outdoor scenarios.

To calculate the distance *D* to an object, the TF Mini uses Equation (1):

(1) $$D=\frac{c}{2}\cdot \frac{1}{2\pi f}\cdot \Delta \varphi ,$$

where *c* is the velocity of light in vacuum, *f* is the modulated signal frequency, and Δφ is the phase shift between the emitted signal and the received signal. The TF Mini is reported to optimize various compensation algorithms and can send ranging information up to 1000 times/s (i.e., 1 time/ms) [19]. Its ranging method along with the high update rate is expected to be sufficient for people with vision impairment, who usually have a lower movement speed (about 0.72 m/s as reported in [20]). As such, the estimated distance given by the TF Mini is expected to be reliable.

**Figure 5:**
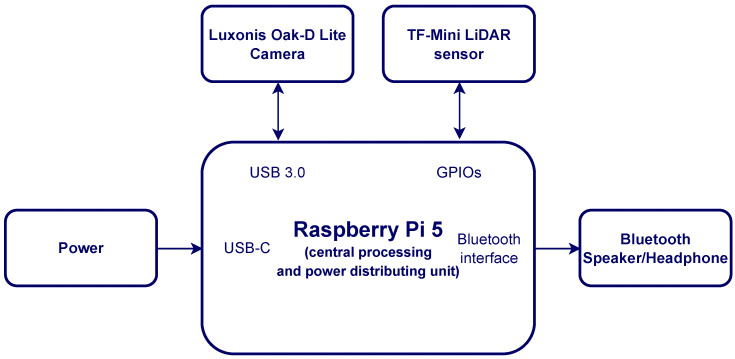
Schematic diagram of VAS.

**Figure 6:**
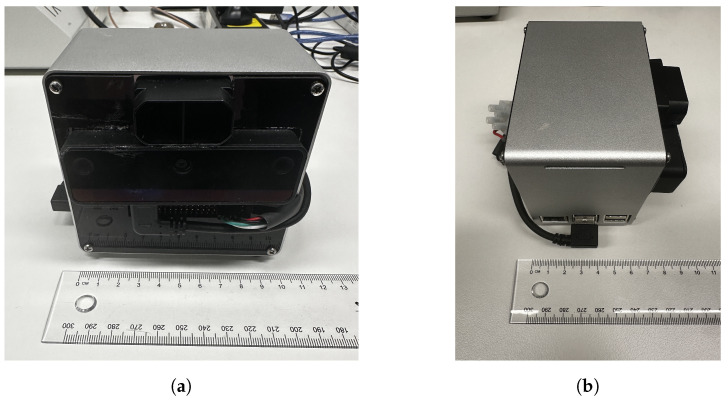
Complete prototype of VAS with its (**a**) front view and (**b**) side view.

To ensure the compactness of VAS, all hardware components are assembled into a single unit. The Oak-D Lite is connected to the Raspberry Pi 5 via a USB 3.0 port, and the DepthAI library is leveraged to establish the communication. The TF-Mini is connected to the Raspberry Pi 5 via General-Purpose Input/Output (GPIO) pins. During debugging and indoor experiments, VAS is powered by the standard 27 W USB-C power supply adapter for Raspberry Pi 5, while Bluetooth devices like earphones or speakers are connected via the Bluetooth interface of the Raspberry Pi to deliver alert messages. Figure 5 shows the schematic diagram of physical components in VAS. The total cost to produce VAS is about A$527 (equivalent to US$364). The final dimension of VAS (without battery) is approximately 10.5 cm × 9 cm × 7 cm, and the final weight (without battery) is 600 g, which means VAS is extremely portable. The current form factor has this size due to our available aluminium case, but it could be reduced to, for example, 10.5 cm × 7 cm × 7 cm, if a smaller case is used. VAS’s weight can also be further reduced if a plastic case is used. The TF Mini is rated IP65 [19], which means that it is water and dust resistant, while the USB connections between the Oak-D Lite and the Raspberry Pi can be made waterproof by utilizing inexpensive commercial rubber plugs and gasket seals. The complete front view and side view of VAS are shown in Figure 6a,b.

### 2.2. Software and Algorithms

#### 2.2.1. ROS 2 Backbone and Architecture

VAS is structured on the Humble distribution of ROS 2 (Robot Operating System 2)—an open-source robotics middleware [21]. VAS utilizes the Publisher-Subscriber (PubSub) model, services, and message-passing mechanisms of ROS 2 to coordinate real-time communications between processing nodes containing sensors and the TTS feedback system. The system is deployed on Debian GNU/Linux 12 (Bookworm) running on the 8 GB RAM Raspberry Pi 5. Different components of VAS, including the Oak D-Lite and the TF Mini, are built in independent ROS 2 nodes. These nodes communicate seamlessly using the PubSub model, where one node (for example, the TF Mini node) publishes its data to a topic, and other nodes (for example, the VAS Core node) subscribes to that topic. However, this leads to an excessive queuing, as such, the PubSub model must have a Quality of Service (QoS) setting to ensure that data is delivered based on its importance, providing a reliable and real-time performance. VAS leverages two QoS mechanisms. The first mechanism is Best Effort QoS used for non-critical data, such as image data from the Oak D-Lite, where occasional frame losses will not significantly affect the system performance. The second mechanism is Reliable QoS, which ensures that critical data, such as obstacle detection results and distance measurements, is always transmitted and processed.

**Figure 7:**
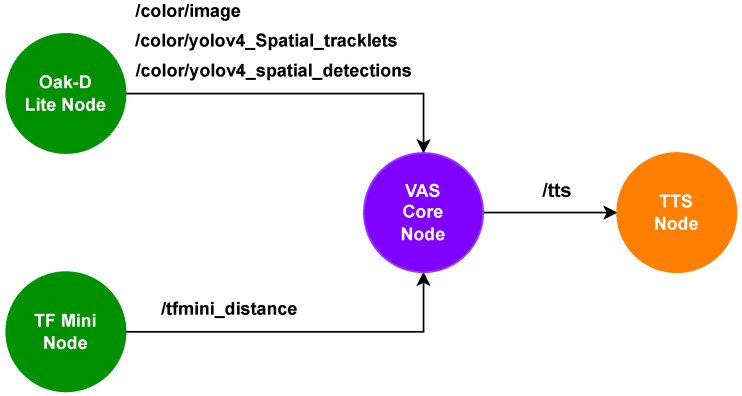
ROS 2 nodes and topics in VAS.

The main ROS 2 nodes and topics in VAS are described in Figure 7. In VAS, the TF Mini node initializes the serial port at 115,200 bauds [18] for an efficient data transfer. It is responsible for reading distance measurements (up to 12 m) from the TF Mini and publishing the data as Float32 messages to the /tfmini_distance topic. The data flow of this node is optimized by logging results every 100 ms to minimize excessive logging and prevent performance degradation. To ensure invalid data filtering, this node filters out erroneous data such as unreliable strength measurements or distances beyond the valid range (for example, over 12 m). Thus, only accurate data is processed.

**Figure 8:**
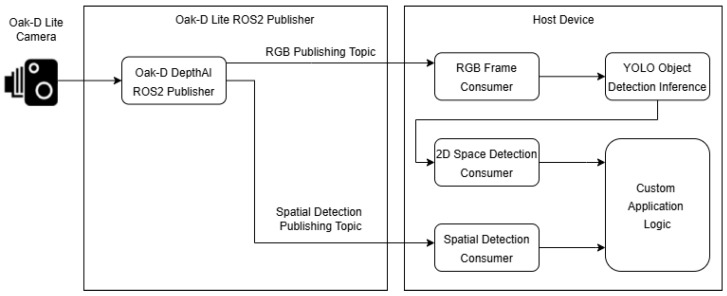
Baseline publishing diagram of the Oak-D Lite node (adapted from [22]).

The Oak-D Lite node in VAS is based on [22], as shown in Figure 8. On the Oak-D Lite, the YOLOv4-tiny algorithm processes image data and performs object detection tasks. The topics /color/image and /color/yolov4_spatial_detections are derived from the baseline publishing topics, which are the RGB Publishing topic and the Spatial Detection Publishing topic in Figure 8. Besides, the /color/yolov4_Spatial_tracklets topic is realized by enabling the ObjectTracker module in the DepthAI pipeline, assigning tracking IDs to consecutive detection frames from the spatial detection output, which is suitable for a reliable and stable TTS mechanism. The internal Stereo Depth function calculates the depth map using stereo vision from the stereo left and right cameras, and inputs the result to the YoloSpatialDetectionNetwork to calculate 3D position (x, y, z) of objects. The network then publishes object detection results with spatial coordinates in the spatial detection topic, providing both object recognition and spatial awareness. By doing this, the Oak-D Lite node can precisely provide both classification and distance information of each detected object. For error handling, the node also filters out low confident detection results (those with confidence score under 0.5) and assigns tracking IDs to ensure reliable and consistent feedback for users.

The VAS Core node processes object detection and object distance data from the Oak D-Lite node and the TF Mini node to generate alarming feedback messages. This node continuously listens to four key data streams: images (from the /color/image topic), object detection tracklets (from the /color/yolov4_Spatial_tracklets topic), object detection results (from the /color/yolov4_spatial_detections topic) and object distances (from the /tfmini_distance topic). Next, it filters the detected objects using a predefined map of common objects (similar to the Common Objects in Context (COCO) dataset). Then, it calculates the relative angles and positions of objects to the user. To handle with errors, this node logs and tracks missing or invalid data (e.g., lack of image or distance input) and resets counters after multiple errors. The VAS Core node discards invalid objects, for example, those with zero depth or fall outside the filter map. It then creates alarming messages depending on the urgency level and publishes these messages to the /tts topic, to which the TTS node subscribes and converts into voice feedback for the user.

The TTS node is responsible for converting the latest alarming messages from the VAS Core node into WAV audio files using Piper—a neural TTS system suitable for Raspberry Pi [23]. Sound eXchange (SoX) audio processing tool is utilized to speed up the playback by 1.5x to ensure the timely delivery of alarming feedback to the user and optimize the system interaction with changing environments without sound interruptions. SoX also helps with noise and silence removal [24]; thus, reducing the latency of TTS messages. The TTS node implements two separate threads—one for generating audio files and the other for playing them back in sequence, ensuring a smooth audio output. The queue is ensured to have a maximum size of one, so only one message is processed and played at a time, preventing generated audio files from overwhelming the system or causing backlog. During our experiments, the recorded publishing rate of topic /tts and /tfmini_distance is approximately 10 Hz, while that of /color/yolov4_Spatial_tracklets, /color/yolov4_spatial_detections, and /color/image is approximately 19 Hz. This means the processing latency of the system is about 100 ms, which ensures a real-time response to the environment.

#### 2.2.2. Docker Containerisation and YOLOv4-Tiny Algorithm

**Figure 9:**
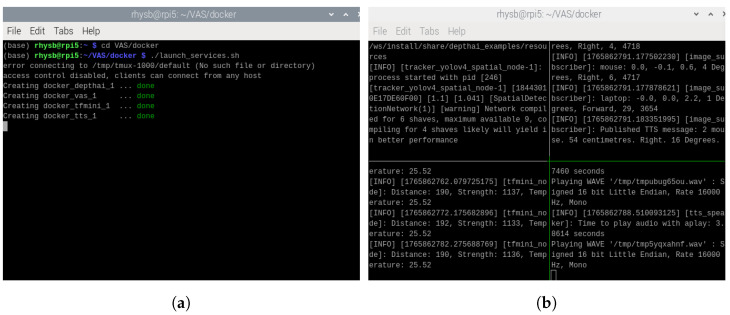
(**a**) Initialization of containers and (**b**) operation of containers in VAS.

**Figure 10:**
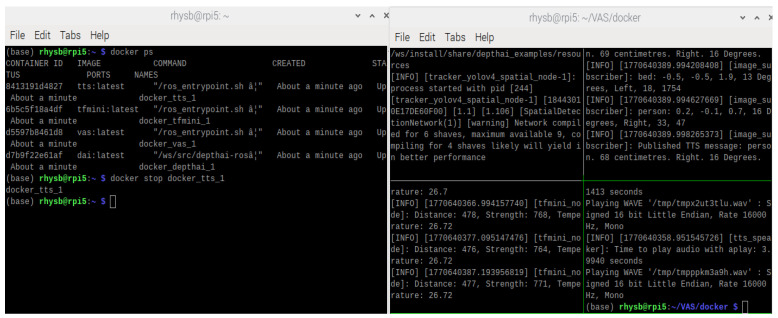
The stable operation of the system regardless of a stopped container.

Docker is a platform that allows applications and their dependencies to be packaged into containers [25]. Four separate containers are built in VAS as shown in Figure 9. In Figure 9b, the top-left container is docker_depthai_1, the top-right container is docker_vas_1, the bottom-left container is docker_tfmini_1, and the bottom-right container is docker_tts_1. Each container can run, update, or restart independently; thus, even if one container encounters a failure, others remain unaffected. For example, in Figure 10, when the docker_tts_1 container is stopped intentionally, other containers still operate normally. The combination of Docker and ROS 2 in VAS allows containers to communicate through ROS 2 topics, facilitating the seamless flow of data. This also allows the system to be highly modular and scalable. In addition, with Docker Compose, all containers are orchestrated and launched together using a simple configuration file, ensuring that containers are initialized with proper settings in one step. In VAS, Docker version 20.10.4 is used, while Docker Compose version v2.35.1 is utilized.

The YOLOv4-tiny algorithm is the minimized version of the YOLOv4 algorithm [26]. It is trained on the COCO dataset, which is a common dataset with 80 labels for 80 common objects in daily life. In VAS, the YOLOv4-tiny algorithm is run on the Oak-D Lite; thus, object detection is performed directly on this camera, reducing AI processing tasks for the Raspberry Pi 5. The YOLOv4-tiny model looks at an input image at two detection scales of 26 × 26 and 13 × 13 to detect objects of different sizes [12]. It is suitable for VAS because it allows fast and real-time object detection, and generates lighter GPU and CPU load. The YOLOv4-tiny uses the backbone CSPDarknet53-tiny with fewer feature extraction layers in the CSPBlock module than the CSPDarknet53 [12]; thus, increasing the speed of object detection. However, this comes with a trade-off, as the accuracy of detection might be lower when the tiny model cannot analyse the input images in as many details as the full model in some cases.

#### 2.2.3. Vision Alarming Algorithm

An algorithm named Vision Alarming Algorithm running on the VAS Core node is developed. It collects and processes input data from the TF Mini and the Oak-D Lite, then creates the output alarming messages containing critical information on object classification, distance, direction, and relative angle. Next, it publishes these messages to the TTS system to be converted to voice alerts for the user.

At the beginning, the algorithm subscribes and continuously listens to the four topics from peripheral devices of VAS, which are /color/image, /color/yolov4_Spatial_tracklets, /color/yolov4_spatial_detections, and /tfmini_distance.

To process incoming data, the algorithm first defines a general alarming message structure based on the distance information from the TF Mini if this data is valid. This means if no object detection happens at the Oak-D Lite, the system can still generate alerts based on the valid distance data from the TF Mini. This becomes crucial in dark conditions as VAS can still warn the user about obstacles ahead. Otherwise, if object detection results by the camera are available, then these information sources are combined and compared with the distance information provided by the TF Mini to output detailed and more precise alarming messages to the user.

With each object detection result above the score threshold of 0.5 from the spatial tracklets topic, the algorithm repeatedly extracts object’s label, tracking ID, tracking age, and 3D position. Based on the x- and z-coordinates of the object, it calculates the relative angle and decides the relative direction of the object to the user. To help with debugging and better evaluation of the system performance, the spatial tracklets topic is used in parallel with the spatial detection topic, whose raw detection results are used to draw bounding boxes with real-time confidence scores on the latest images obtained, using the OpenCV 4.12.0 tool.

**Figure 11:**
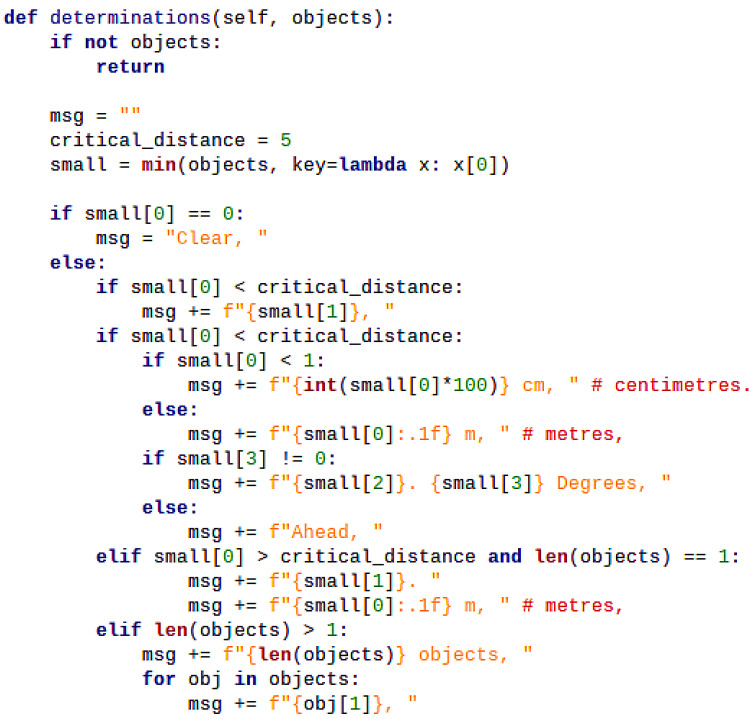
Code snippet for the determination logic of the Vision Alarming Algorithm in VAS.

Depending on the urgency level of approaching objects, the algorithm determines what to insert into the TTS messages, with the logic shown in Figure 11. The urgency of approaching objects is classified into two levels, separated by a threshold of 5 m, which can be easily adjusted. In case objects are within 5 m in front of the system, if the distance to the nearest object is smaller than 1 m, the algorithm will notify the distance information in centimeters. In addition, the algorithm will categorize its direction as “Ahead”, “Forward”, “Right” or “Left”, depending on the calculated relative angle. Specifically, “Ahead” is when the object is within 5 m and the (rounded) angle is 0°. This may happen, for example, in a dark condition when there is no data from the Oak-D Lite, and the system merely depends on the TF Mini with its narrow FoV. Another case of “Ahead” is when the object is perfectly in front of the Oak-D Lite, so the angle based on detection results is also 0°. “Forward” is for other cases when objects are still within 5 m and the angle is not 0° but under 2°. Otherwise, the position will become “Right” or “Left”, along with angle values to be informed to the user. In case objects are beyond 5 m, if there is only one object, the TTS messages will announce the object and the distance in meters. If there are multiple objects, the TTS messages will announce the total number of objects, then list each object label without informing the distance to each individual object to reduce the latency of the TTS messages. The classification mechanism of VAS will be mentioned in more detail in both indoor and outdoor experiments in Section 3 below.

**Figure 12:**
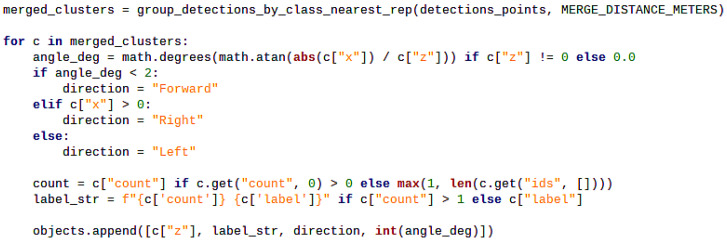
Code snippet for grouping nearby similar objects by the Vision Alarming Algorithm.

To increase the effectiveness of the TTS feedback, especially in high traffic or cluttered environments, a grouping algorithm is proposed, as shown in Figure 12. Here, the distance threshold between two or more objects is set to 1.2 m, which can be easily adjusted. The algorithm will check whether the detected objects have the same label, which means that the objects are of the same type. If so, it calculates the Euclidean distance between them. If this distance is smaller than the threshold, the system will report all these objects as a group in one united message, and warn the user about the distance to the nearer object. For example, if there are two people standing next to each other in front of the system, one is 4 m and the other is 4.5 m away, the system will generate alarming messages saying “2 person, 4 m, Ahead.” This means VAS can notify users by one brief warning message for a group of similar and close objects, allowing it to deal with high traffic or cluttered environments. The more such objects appear in the view of the device, the more significantly the grouping algorithm shortens the processing and speaking time.

Before alarming messages are published as strings to the topic /tts, a filtering mechanism is applied. That is, after a new TTS message is created, it is compared with the last message. If the messages are the same (meaning that this is the same object at the same distance/direction) and the time interval between these messages is still within a certain time window, the new message will not be published. Currently, the time window is set at 4.5 s, which is around three times the time needed to play back a typical TTS message (1.6 s), and this time window value can be changeable. If the new message is not the same within this time window, the system will publish it immediately. Thus, the system will automatically limit the frequency of similar TTS messages to reduce the cognitive load on the user, while still ensuring that every new appearing object can be warned in advance. Accepted TTS messages are then converted into speech and delivered to the user’s paired earphones/speakers via Bluetooth interface. The playback speed is increased by 1.5× through the command in SoX to ensure the timely delivery of those messages.

The pipeline processing of VAS is almost instantaneous in the sense of hearing. When the TF Mini or the Oak-D Lite detects something at a distance of up to 12 m, VAS can generate a warning message within 100 ms. (This generation rate can be as short as 1 ms depending on the update frequency of the TF Mini to be selected). In addition, the system takes no more than 2.7 s to deliver a warning notification to the user. The instant generation of TTS messages along with the rapid delivery of those messages enable early warnings for users to listen and process. Given that VAS is designed for visually impaired people, whose moving speed is typically within the range of 0.65–1.11 m/s [27] and around 0.72 m/s on average [20], which is primarily lower than that of normal people, they can have enough time to process the voice information. With the given walking speed, within 2.7 s, the user can walk for around 1.76–3 m (1.94 m on average). Since the operating range of VAS is up to 12 m, the users still have a safety gap of approximately 9–10 m, which is sufficient for the users to decide to steer their walking direction, slow their movement, or stop walking.

### 2.3. System Workflow

**Figure 13:**
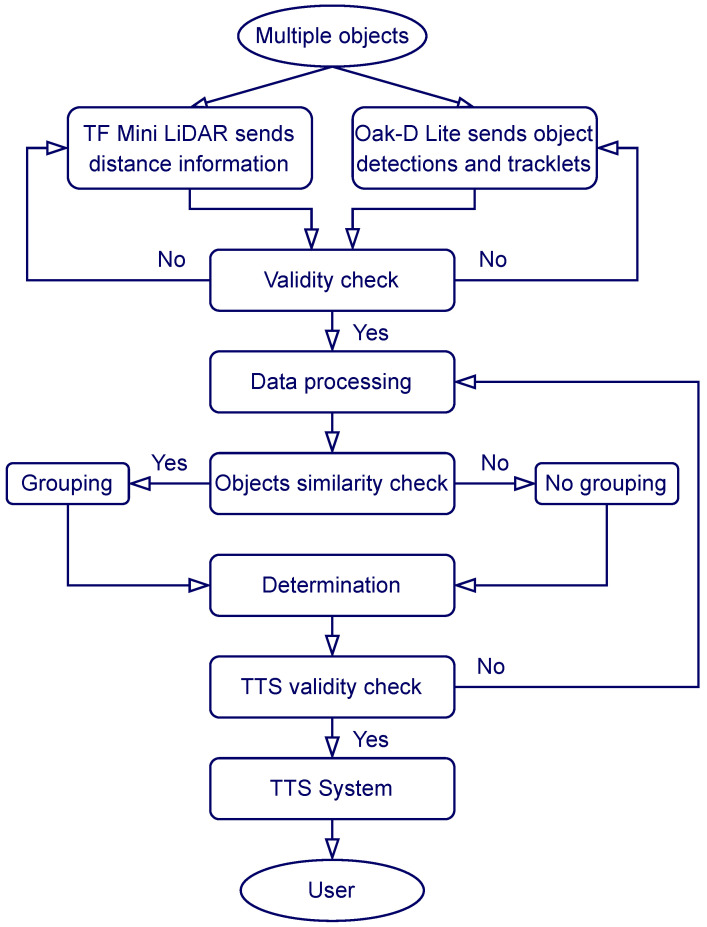
The general workflow of VAS.

The general workflow of VAS is described in Figure 13. First, the TF Mini and the Oak D-Lite provide distance and object detection data, allowing for object recognition and spatial awareness enhancement. Next, the VAS Core node checks data validity, then processes the data by filtering objects belonging to the label map, calculating distances, directions, and relative angles of the objects, grouping nearby similar objects, drawing bounding boxes for testing and debugging, and constructing the output alarming messages containing critical information determined by the urgency level. Following this, the TTS filtering mechanism decides if the newly generated message is valid for publishing within the time window. If the message is valid (i.e., it contains updated information), it is received and converted into speech by the TTS system using the Piper neural network and delivered to the user via Bluetooth devices. This workflow enables a precise, event-triggered, and timely delivery of navigation feedback, with Docker providing flexibility and ROS 2 ensuring seamless communications between system components. Note that the frequency of the audio feedback is an individual-based setting parameter, which can be adjusted based on the speed of TTS and SoX or the threshold distance. Moreover, most of CV processing steps can be optimally done in the Oak-D Lite; thus, facilitating high and accurate object detections and classifications while maintaining a minimal hardware form factor.

## 3. Experiment and Result Analysis

### 3.1. Experimental Setup

To prepare for indoor and outdoor experiments, a Bluetooth earphone is connected to the Raspberry Pi 5, through which the TTS feedback is delivered to the user. When vision-impaired users move, they will wear a headphone/earphone, and either carry the powered VAS, attach it to the wheelchair, or wear it around their waist to easily capture pictures and videos in indoor and outdoor environments. In our experiments, a portable monitor is connected via an HDMI cable to the Raspberry Pi 5. It is important to note that the monitor is removed in the daily usage of VAS. It is only connected in our experiments to visualize the operating processes of VAS testing.

Indoor testing was first conducted in a laboratory office with sufficient light, insufficient light, or near-dark conditions and with common objects such as laptop, bottle, keyboard, and mouses within 2 m from the system, which resembled the real condition of an office. The corresponding average light intensities were 392 lux, 16.3 lux, and 0.0 lux, respectively, measured by a S8608 light meter, where 0.0 lux corresponds to the minimum readable value of the device, indicating significantly low light intensity. In addition, to evaluate VAS applicability in daily situations, further experiments were conducted in a bedroom during daytime and at night, under the average light intensities of 200.8 lux and 67.2 lux, respectively.

Outdoor testing was conducted in the main campus of the University of Wollongong, Australia, with terrain-changing paths, people, and vehicles appearing in different directions, which resembled the real-life outdoor traffic navigation scenarios. To comprehensively evaluate the system performance, experiments were performed in different conditions, including sunny days and at nights. The average light intensities for these scenarios were 41,136.7 lux (unshaded conditions), 2000.1 lux (shaded conditions), and 17.7 lux (nighttime conditions), respectively. During outdoor experiments, the experimenter occasionally moved faster to test the camera ability in detecting objects in case the input video was blurry. Given that the average walking speed of a vision-impaired person is around 0.72 m/s [20], the normal (or fast) walking speed in our experiments refer to the speed of around 0.9 m/s (or 1.3 m/s), respectively. Two metrics, namely the precision of object detection and the reliability of TTS messages, were used to evaluate the system performance. Precision was based on the average confidence score of object detection results. As mentioned in Section 2.2.1, only detections with the confidence score of above 0.5 were accepted. To evaluate the reliability of TTS messages, the clarity, timeliness, and correctness of those alarming messages were considered. It was expected that the TTS system could seamlessly and precisely provide critical information to the user, including the object type, distance, direction, and relative angle depending on the level of urgency.

### 3.2. Results and Analysis

**Figure 14:**
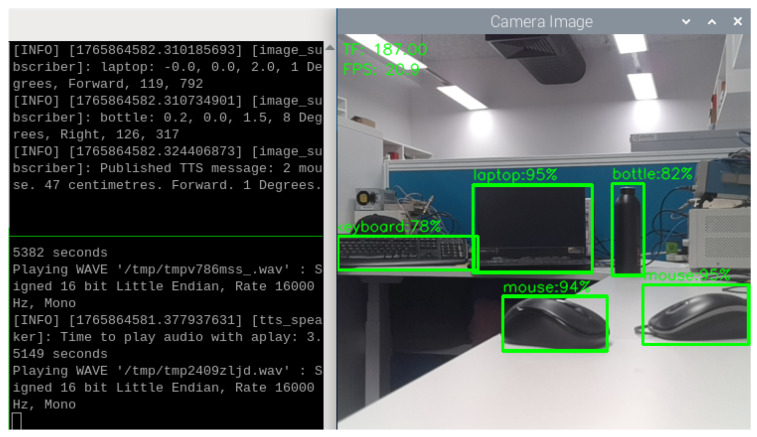
Object detections and TTS results in a laboratory office with sufficient light.

Practical indoor testing of VAS shows promising results in all sufficient, insufficient and no light conditions. For example, in Figure 14, in a laboratory office with sufficient light, VAS is able to detect the laptop, mouse 1, mouse 2, keyboard, and bottle with the confidence scores of 95%, 95%, 94%, 78%, and 82%, respectively. VAS can also continuously generate TTS feedback describing the most urgent objects, and perform object grouping in case two or more objects of the same type are present. In this situation, VAS fuses the distance information from both the TF Mini and the Oak-D Lite to determine the TTS feedback and to provide warnings about the most urgent (the nearest) obstacles. That means the most urgent objects (among detected obstacles) are the ones that are nearest in distance and within the FoV of VAS, which is also the moving direction of the users. In can be seen from Figure 14 that, even the distance data from the TF Mini is 187 cm, VAS uses the stereo depth data from the Oak-D Lite to indicate that there are much closer objects. In addition, as there are two mouses nearer to the system, it continuously generates the TTS messages “2 mouse, 47 cm, Forward, 1 Degree.” Other objects, such as the laptop, bottle, and keyboard, are still identified, but they are less urgent within 5 m according to the alarming mechanism of VAS; thus, they are not mentioned in the TTS messages.

**Figure 15:**
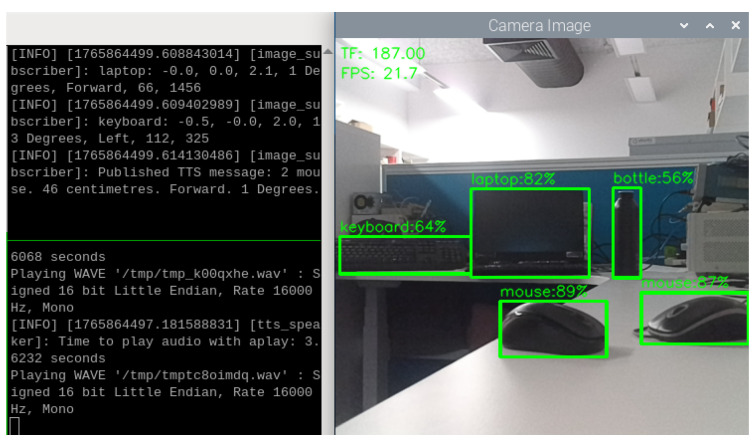
Object detections and TTS results in a laboratory office with insufficient light.

When lighting becomes insufficient, as shown in Figure 15, VAS is still able to detect the same objects with the confidence scores of 82%, 87%, 89%, 64%, and 56%, respectively. It could be observed that lighting conditions may have a considerable impact on the performance of the YOLOv4-tiny model in the Oak-D Lite. In this case, the confidence scores of the keyboard and bottle drop by 14% and 26%, respectively, while those of the mouse 1 and mouse 2 decrease by 8% and 5%. Nevertheless, VAS can still detect objects and generate TTS feedback reflecting the positions and distances of these objects. That means VAS can still produce the TTS messages as “2 mouse, 48 cm, Forward, 1 Degree.” with only a minor difference in the distance information compared to the previous scenario of sufficient light. The performance of VAS in both strong lighting and weak lighting conditions have demonstrated that the integration of the Oak-D Lite is useful since it can function well in a real-time assistive system even in a weak lighting condition. Another important observation is that the distance data measured by the TF Mini is unaffected by the change of lighting conditions in the environment, suggesting its potential to help VAS perform properly in worse lighting or even dark conditions.

**Figure 16:**
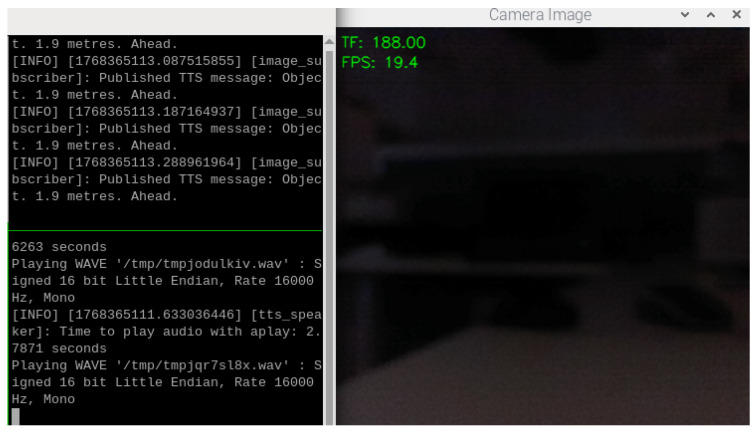
Object detections and TTS results in a laboratory office with no light.

To further evaluate the ability of VAS in different scenarios, all lights in the laboratory office are then switched off. The result given in Figure 16 shows that when the room is dark, the generated TTS messages are “Object, 1.9 m, Ahead.” This validates the ability of VAS to function even when there is almost or completely no light. Thanks to the TF Mini, which still works properly in dark conditions, VAS can still provide users with alarming feedback to help them avoid collisions with obstacles ahead. It is important to note that, in this case, the Oak-D Lite cannot perform object detection due to the no light condition; thus, the system merely relies on the TF mini, so the TTS messages are generated based on the distance readings from the TF Mini to the obstacles appearing in its FoV. Furthermore, the TTS results in all the above experiments have confirmed that the position classification logic is consistent with the proposed algorithm mentioned in Section 2.2.3.

**Figure 17:**
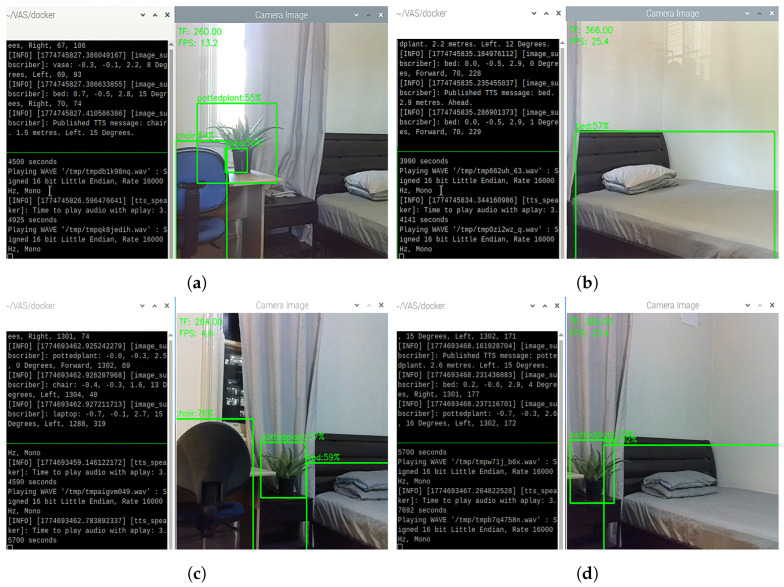
Object detections and TTS results in the case of (**a**,**b**) a bedroom at daytime and (**c**,**d**) a bedroom at nighttime.

In Figure 17a,b, in a bedroom with medium daytime light, VAS is able to detect common bedroom objects, such as chair, potted plant, and bed with confidence scores of 54%, 55%, and 57%, respectively. VAS can also continuously generate TTS feedback that describes the most urgent objects. Noticeably, in Figure 17a, even being affected by the strong sunlight from the window, the system could still operate normally to warn the user, indicating the reliability of VAS against such challenging scenarios.

In Figure 17c,d, in a bedroom at night condition, VAS is able to detect chair, potted plant, and bed with the confidence scores of 76%, 57%, and 59%, respectively. VAS can also continuously generate TTS feedback that describes the most urgent objects. It was noticed that in this scenario, as VAS is no longer affected by the strong daytime light, the system could detect objects with noticeably higher confidence scores. In addition, during these indoor experiments, Docker usage observed on Raspberry Pi varied from 140–220% (out of maximum 400% of 4 cores of Raspberry Pi’s CPU), indicating that the system on average uses 35–55% of the total CPU capacity. These results demonstrated the potential of VAS to provide reliable support for vision-impaired users to avoid collisions with common objects encountered in indoor scenarios, such as in a bedroom.

Outdoor navigation is another primary application of VAS. To carefully examine the system performance in daily outdoor movements, VAS is tested in different conditions, including sunny daytime and nighttime, in either slow movement cases or fast movement cases. Given that VAS is designed for visually impaired users, in most tests, the experimenter walks slowly and steadily. However, to test the auto-focus feature of the Oak-D Lite camera and its object detection capability in blurry cases, the experimenter occasionally walks faster than normal people. With respect to personal privacy, individuals appearing in our pictures are obscured in solid masks.

**Figure 18:**
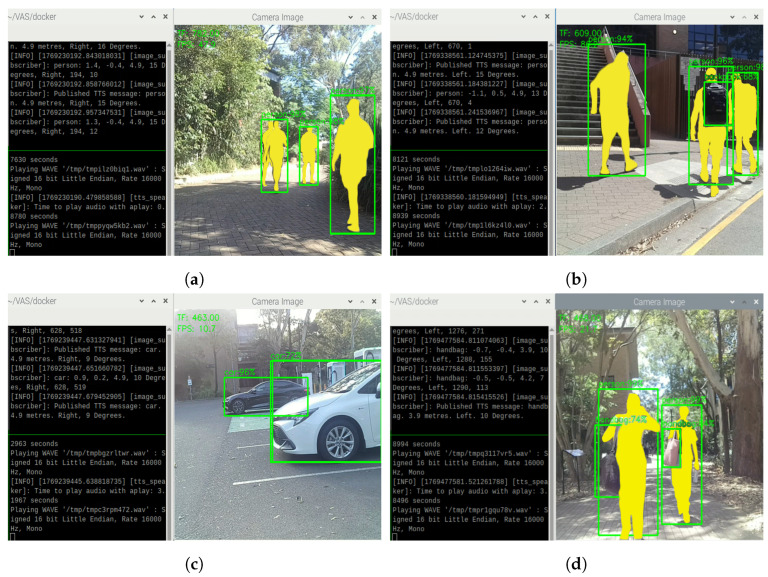
Object detections and TTS results on a sunny day (with no grouping cases) on (**a**) people, (**b**) people, (**c**) cars, and (**d**) handbags.

On sunny days with sufficient sunlight, VAS is able to detect and generate continuous TTS feedback as expected. For example, in Figure 18, VAS is able to detect single objects like person (Figure 18a,b), car (Figure 18c), and handbag (Figure 18d) in different directions and distances with the average confidence score of approximately 90.8%. Following the successful detections, VAS continuously generates TTS feedback, describing the most urgent objects within 5 m to the user. In Figure 18, the nearest objects after calculation are the individuals, the white car, and the handbag, so the system continuously provides alarming messages about them by informing the types of obstacles (i.e., the object labels), distances, directions, and relative angles. The combination of distance information from the TF Mini and the Oak-D Lite is performed in such a way that, if the distance data from both the TF Mini and the Oak-D Lite is available, VAS prioritizes the nearest obstacles to warn the user. In addition, as shown in Figure 18c, even being exposed to the strong sunlight, VAS can still perform object detection and TTS message generation normally, demonstrating a certain level of robustness.

**Figure 19:**
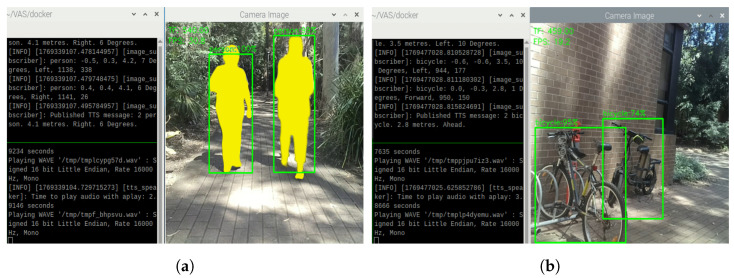
Object detections and TTS results on a sunny day (with grouping cases) on (**a**) people and (**b**) bicycles.

The grouping algorithm of VAS also performs as expected during our outdoor testing. As shown in Figure 19, VAS generates TTS feedback describing the most urgent objects, and grouping the objects in the case where two or more similar objects are present. In this figure, there are two people (Figure 19a) and two bicycles (Figure 19b) that are in front of the system; thus, the system continuously provides alerts about those obstacles by notifying “2 person” or “2 bicycles”, along with their distances, directions, and relative angles. The combination of distance information from the TF Mini and the Oak-D Lite is performed in a similar way as the previous cases.

**Figure 20:**
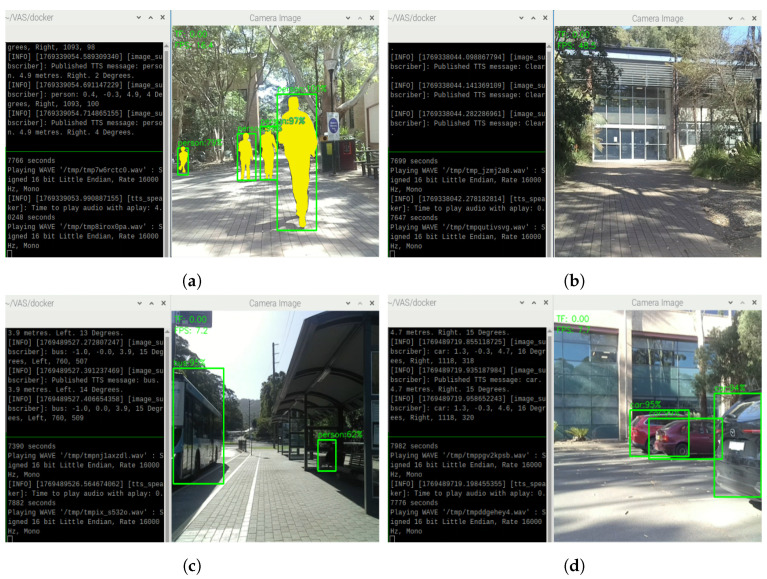
Object detections and TTS results on (**a**) people, (**b**) no obstacle, (**c**) buses, and (**d**) cars in case the TF Mini has no readings.

During outdoor testing, it can be realized that the TF Mini might sometimes struggle when the distance from the object appearing in its FoV is above its maximum range of 12 m. In those cases, the reflected laser signal is insufficient for precise distance calculations; thus, the TF Mini reading will return 0. In this case, if the Oak-D Lite does not detect obstacles either, VAS will notify “Clear.” in its TTS messages, as shown in Figure 20b. If the Oak-D Lite detects obstacles, then the data from the Oak-D Lite is processed. After that, the critical information about those objects will be informed in the TTS messages as expected, as shown in Figure 20. In this figure, the nearest objects are person (Figure 20a), bus (Figure 20c), and black car (Figure 20d); thus, the system provides alerts about them by reporting their object label, distance, direction, and relative angle. In addition, as shown in Figure 20c, regardless of the strong sunlight, VAS can still detect the bus and the person in distance, demonstrating the system’s reliability in challenging scenarios. It is important to mention that the FoV of the TF Mini is narrow; thus, some objects left or right ahead might be out of its detection range. However, the TF Mini is crucial in the case that straight forward objects are approaching the user and in the case of dark conditions, as proved in the indoor situations mentioned previously and the outdoor scenarios to be mentioned below.

**Figure 21:**
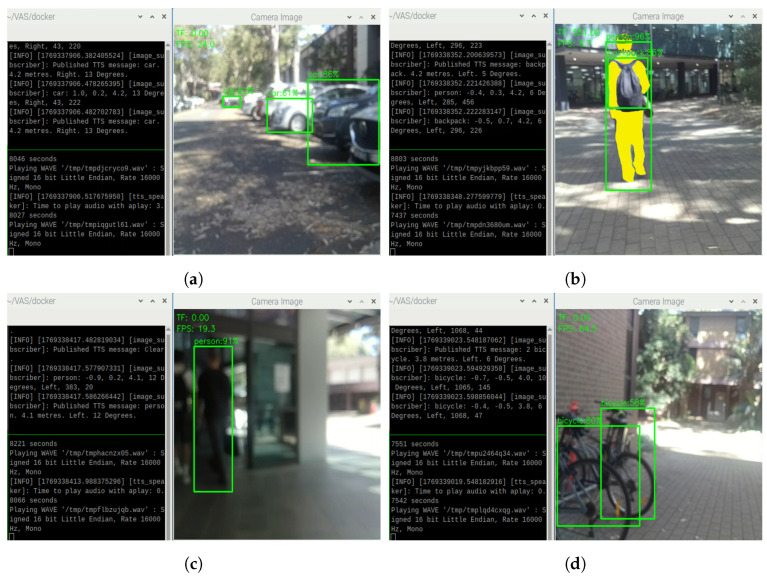
Object detections and TTS results on (**a**) cars, (**b**) backpack, (**c**) people, and (**d**) bicycles in case of blurry camera.

To challenge the auto-focus feature of the camera, the object detection ability of VAS, and the TTS capability in blurry cases, the experimenter occasionally walks faster than normal people during outdoor testing. It is realized that the camera occasionally struggles to remain in focus in some situations. When the camera becomes out of focus, it might take a few seconds to gain auto-focus properly. The object detection and grouping logic thus can be affected. Nevertheless, the object detection and TTS generation features of VAS can still perform reasonably well, as shown in Figure 21. In these cases, VAS can detect cars (Figure 21a), person (Figure 21b,c), backpack (Figure 21b), and bicycles (Figure 21d) with the average confidence score of 77.5%.

**Figure 22:**
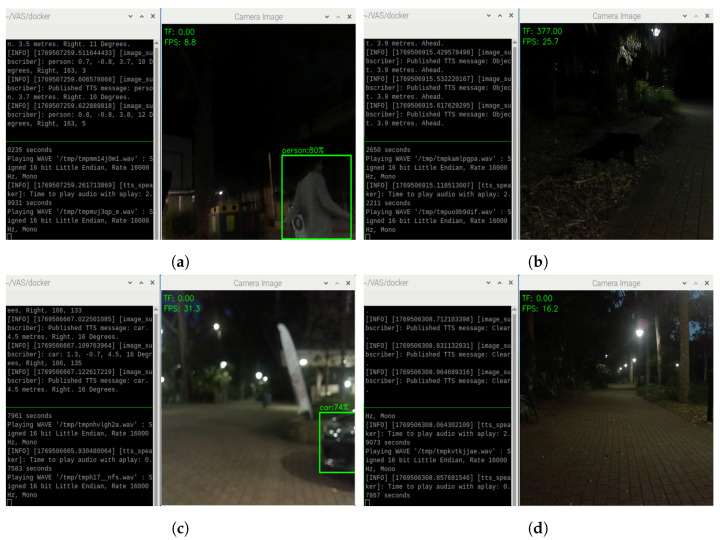
Object detections and TTS results for the case of (**a**) people, (**b**) unrecognized objects, (**c**) cars, and (**d**) no obstacle in a night outdoor condition.

In night outdoor conditions, VAS is still able to detect people, obstacles, and generate TTS alarming messages, as shown in Figure 22. During nighttime, if there is some lighting, VAS can perform object detection and TTS generation, but at a lower confidence score compared to the daytime cases, as shown in Figure 22a,c. When there is no object or obstacle detected by the TF Mini and the Oak-D Lite, the system delivers the “Clear” status like in the daytime, as shown in Figure 22d. In case of dark conditions, the system again relies on the TF Mini. For example, as shown in Figure 22b, the Oak-D Lite could not recognize the bench, but the TF mini could still detect its presence; thus, the generated TTS messages are “Object, 3.9 m, Ahead.”

All the outdoor testing results have proved the potential of VAS in providing reliable support for vision-impaired users to avoid collisions with pedestrians, bicycles, vehicles, and other obstacles, even in crowded urban areas in various conditions. Outdoor testing was conducted several times a day. For each time, the experimenter carried VAS and walked around the university campus for approximately one hour. This has further demonstrated the stable operation of the system in outdoor navigation applications. During indoor and outdoor tests, the recorded power consumption of VAS varied from 12–15 W on average, corresponding to the energy consumption of 12–15 Wh over one hour of operation.

Table 3 summarizes the performance of VAS in indoor office and outdoor scenarios with the average precision score of object detection results in the Oak-D Lite and the reliability of TTS feedback as evaluation criteria. It can be seen that, in various conditions, VAS is still able to detect objects and provide timely feedback to users with acceptable precision and reliability. Table 4 summarises key performance metrics of VAS during experiments. A video demonstrating the operation of the system in an indoor environment is available at [28].

### 3.3. Discussion

VAS utilizes the Raspberry Pi 5 and the Piper neural TTS system, whose effectiveness has been proven in some existing works, while exploring the potential of the Oak-D Lite in running object detection models and the ability of the TF Mini in detecting obstacles in various scenarios. VAS provides users with real-time audio alerts, which contain critical information about the presence, type, distance, direction, and relative angle of obstacles, allowing users to improve spatial awareness, make timely decisions, and navigate more safely, confidently, and independently in different environments. The main and noticeable features of VAS are described as follows:

- Real-time Object Detection: VAS utilizes the YOLOv4-tiny model running on the Oak D-Lite AI camera to detect a wide range of objects in real-time scenarios. Previous studies such as [29] reported that electronic travel aids, which VAS belongs to, can help vision impaired users achieve improved object detection, larger detection ranges, and larger safety ranges during obstacle avoidance, in comparison with long canes. Traditional long canes also cannot provide information about objects at higher levels (for example, above waist height) or information about object characteristics, as discussed in [30]. This emphasizes VAS advantages compared to traditional long canes.
- Depth Perception: The Oak D-Lite camera provides stereo depth perception, while the TF Mini adds highly accurate distance readings for objects directly ahead.
- Audio Feedback: A TTS system converts alarming messages with critical information regarding object detection, object distance, and position into easy-to-understand spoken alerts for the user.
- Modular Design: Each sensor or component of VAS operates within its own container, allowing easy upgrades and modifications.
- Standalone Design: VAS does not require the Internet or a GPS connection to operate. The system is designed as a plug-and-play solution; thus, optimizing the convenience of users.
- Cost-effective Design: VAS leverages open-source, cost-effective hardware, middleware, and software, demonstrating its advantage as a relatively low-cost but effective navigation solution.

**Figure 23:**
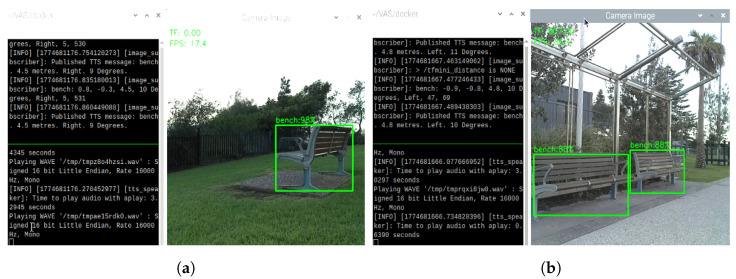
Object detections and TTS results for the case of (**a**) a bench in the park and (**b**) a bench along the footpath.

With the integration of various modular, smart, and cost-effective components, VAS is able to provide support for users in daily fundamental moving situations, from indoor to outdoor, in either light or dark conditions; thus, considerably improving the safety and mobility of its users. Potential applications of VAS include the daily movement, traffic participation, and navigation, as specified below:

- Outdoor Navigation: VAS can potentially help users avoid collisions with obstacles when participating in outdoor traffic, with an effective working range of up to 12 m.
- Indoor Navigation: VAS is expected to be useful for avoiding furniture, walls, and other static obstacles when navigating within buildings or indoor environments.
- Low Visibility Navigation: Even in low-light or no-light conditions, VAS still shows the potential to provide consistent, reliable feedback on the presence of obstacles ahead.
- Active Object Localization: VAS may help vision-impaired users actively locate the object or destination of interest like public facilities. In such cases, users can turn the system 360°. If the targeted object is within the working range of VAS, the system can announce to the user about the presence of that object. One example of this scenario is demonstrated in Figure 23a,b where the user can actively locate the bench in the park and along the footpath.

During indoor and outdoor experiments, it is observed that lighting conditions in the environment, as well as the angle, size, and moving speed of objects can affect how they can be detected and notified by VAS. This first VAS proof-of-concept version still has certain limitations that can be improved in the future. First, we use the YOLOv4-tiny model. This model is optimized for fast object detection; thus, it is suitable for real-time assistive applications like VAS, but the accuracy of detection results might be lower compared to the YOLOv4 full model. As a result, some small objects can be missed or wrongly identified. More powerful object detection models can offer a better performance. In addition, the current AI performance of VAS is based on and limited by the Oak-D Lite with 4 TOPS. To further improve this, one possible solution is to use a Raspberry Pi 5 with an AI HAT (Artificial Intelligence Hardware Attached on Top) offering up to 26 TOPS, which is expected to significantly boost the system overall performance. To provide more reliable warnings, the future hardware version will also consider the integration of ultrasonic sensors or photoelectric sensors to allow for the reliable detection of transparent objects. Furthermore, as regulated by Australian laws, experiments with guide dogs and vision impaired patients require special licenses and approvals from authorities, which were inaccessible to the research team at the time we performed our experiments. Our future work will carry out further comprehensive comparisons with the assistance of the visual impaired researcher in the team (and multiple vision-impaired participants if applicable) to comprehensively compare VAS with other aides, such as guide dogs, smart canes, and traditional canes.

A potential issue for any voice-based assistive device is the heavy cognitive load on the users, meaning that the users need to process a large amount of continuous warning messages, retain the information to keep track of changes over time and make decisions on the perceived information, and possibly confuse if the voice keeps playing continuously. VAS has been designed to reduce this cognitive load through the tandem use of our novel techniques, which include (a) the prioritization of the closest objects, (b) the TTS filtering mechanism, (c) the grouping algorithm, and (d) the brief-but-informative TTS message structures. As a result, the users are provided with necessary, rather than overabundant, information on the obstacles in a timely manner. In the future, we will continue to optimize the TTS message structure to further reduce the cognitive load that users may have when using the system.

## 4. Conclusions

In this article, a real-time, plug-and-play, and cost-effective AIoT vision alarming system is proposed to assist visually impaired people. The system is developed on a Raspberry Pi 5, incorporating the Oak-D Lite AI camera and the TF-mini LiDAR sensor. It also leverages ROS 2, Docker for structuring system components, the YOLOv4-tiny algorithm for object detection, and the Piper TTS system for voice feedback generation. In operation, the system continuously captures the surrounding environments, then performs both object detection and distance and angle calculation. It then creates short, critical voice alert messages and notifies users about the most urgent objects and their relative distances and positions. A visually impaired person is involved as an active member in the analysis, design, and investigation processes, which allows our research team to develop a fit-for-purpose assistive system for vision-impaired people. Experimental results demonstrate the system ability to support users in daily indoor and outdoor navigation situations with a high accuracy of object detection and reliable voice feedback, even in dark conditions or in case of fast movements.

In future work, to address current limitations, we will consider training a custom object detection model to extend the object dataset, utilizing more powerful AI models, exploring other hardware options like the AI HAT to further improve the efficiency of VAS, and optimizing the mechanism of generating alarming messages to enhance the system performance in more sophisticated navigating situations that users may face in real life. We might also consider exploring other ML approaches, e.g., compact DL models [31] and ensemble methods [32], as well as the possible integration between VAS and a Brain-Computer Interface (BCI) device, e.g., the one in [33], to automate the transition from the user’s traffic perception to device control capability, for example, to change the direction of movement of the wheelchair when obstacles appear.

## Figures and Tables

**Table 1:** A qualitative comparison of VAS with previous work.

Paper	Reduction of User Training	Lightweight/ Compactness	GPS Requirement	Sound/ Tactile Feedback	Internet Connection Independence	Objects Variety
[12]	Yes	Yes	Partial	Yes	No	No
[11]	Partial	Yes	Partial	Yes	Partial	Yes
[10]	Partial	Partial	Partial	Yes	Partial	Partial
[9]	Partial	Yes	No	Yes	Yes	Yes
[6]	Partial	Partial	Partial	Yes	Yes	Partial
This paper	Yes	Yes	No	Yes	Yes	Yes

**Table 2:** Main technical specifications of the TF Mini-Plus LiDAR sensor.

Description	Parameter Value
Operating range	0.1–12 m
Accuracy	±5 cm (0.1–6 m)
	±1% (6–12 m)
Measurement unit	cm
Distance resolution	1 cm
Field of View (FoV)	3.6°
Frame rate	1–1000 Hz (adjustable)

**Table 3:** Evaluation of VAS performance in indoor and outdoor scenarios. The Oak-D Lite only works with lighting conditions; thus, precision score is not applicable in no light scenarios.

Scenario	Precision	Reliability
Indoor office (sufficient light)	88.8%	High
Indoor office (insufficient light)	75.6%	High
Indoor office (no light)	N/A	High
Outdoor (sunny)	90.8%	High
Outdoor (sunny-blurry)	77.5%	Acceptable
Outdoor (night)	77%	High

**Table 4:** Summary of performance metrics of VAS during experiments.

Metric	Value
Processing latency	100 ms
Power consumption	12–15 W
Docker usage	35–55% CPU
Highest localization accuracy	5 cm

## Data Availability

Dataset available on request from the authors.

## Abbreviations

The following abbreviations are used in this manuscript:

AI	Artificial Intelligence
AI HAT	Artificial Intelligence Hardware Attached on Top
AIoT	Artificial Intelligence of Things
BCI	Brain Computer Interface
CNN	Convolutional Neural Network
COCO	Common Objects in Context
CV	Computer Vision
DL	Deep Learning
FoV	Field of View
GPIO	General-Purpose Input/Output
GPS	Global Positioning System
GSM	Global System for Mobile Communications
HDR	High Dynamic Range
IoT	Internet of Things
LiDAR	Light Detection and Ranging
LSTM	Long Short-Term Memory
ML	Machine Learning
NoIR	No Infrared Filter
PubSub	Publisher-Subscriber
QoS	Quality of Service
RGBD	Red, Green, Blue, and Depth
ROS 2	Robotic Operation System 2
SoX	Sound eXchange
SSD	Single Shot Detector
ToF	Time of Flight
TOPS	Trillion Operations Per Second
TTS	Text-To-Speech
VAS	Vision Alarming System
VGG16	Visual Geometry Group 16-layer
VPU	Vision Processing Unit
WHO	World Health Organization
YOLO	You Only Look Once
